# Error sensitivity of a log file analysis tool compared with a helical diode array dosimeter for VMAT delivery quality assurance

**DOI:** 10.1002/acm2.13051

**Published:** 2020-10-23

**Authors:** Philipp Szeverinski, Matthias Kowatsch, Thomas Künzler, Marco Meinschad, Patrick Clemens, Alexander F. DeVries

**Affiliations:** ^1^ Institute of Medical Physics Academic Teaching Hospital Feldkirch Feldkirch Austria; ^2^ Private University in the Principality of Liechtenstein Triesen Liechtenstein; ^3^ Department of Radio‐Oncology Academic Teaching Hospital Feldkirch Feldkirch Austria

**Keywords:** ArcCHECK, LINACWatch, log file, VMAT delivery quality assurance

## Abstract

**Purpose:**

Integrating log file analysis with LINACWatch*®* (*LW*) into clinical routine as part of the quality assurance (QA) process could be a time‐saving strategy that does not compromise on quality. The purpose is to determine the error sensitivity of log file analysis using LINACWatch*®* compared with a measurement device (ArcCHECK*®, AC*) for VMAT delivery QA.

**Materials and methods:**

Multi‐leaf collimator (MLC) errors, collimator angle errors, MLC shift errors and dose errors were inserted to analyze error detection sensitivity. A total of 36 plans were manipulated with different magnitudes of errors. The gamma index protocols for AC were 3%/3 mm/Global and 2%/2 mm/Global, as well as 2%/2 mm/Global, and 1.5%/1.5 mm/Global for LW. Additionally, deviations of the collimator and monitor units between TPS and log file were calculated as RMS values. A 0.125 cm^3^ ionization chamber was used to independently examine the effect on dose.

**Results:**

The sensitivity for AC was 20.4% and 49.6% vs 63.0% and 86.5% for LW, depending on the analysis protocol. For MLC opening and closing errors, the detection rate was 19.0% and 47.7% for AC vs 50.5% and 75.5% for LW. For MLC shift errors, it was 29.6% and 66.7% for AC vs 66.7% and 83.3% for LW. AC could detect 25.0% and 44.4% of all collimator errors. Log file analysis detected all collimator errors using 1° detection level. 13.2% and 42.4% of all dose errors were detected by AC vs 59.0% and 92.4% for LW using gamma analysis. Using RMS value, all dose errors were detected by LW (1% detection level).

**Conclusion:**

The results of this study clearly show that log file analysis is an excellent complement to phantom‐based delivery QA of VMAT plans. We recommend a 1.5%/1.5 mm/Global criteria for log file‐based gamma calculations. Log file analysis was implemented successfully in our clinical routine for VMAT delivery QA.

## 1 INTRODUCTION

Volumetric modulated arc therapy (VMAT) is widely spread in modern radiotherapy and was first mentioned in literature in 1995.^1^ The gantry continuously moves around the patient, and the field shape as well as the dose output is changed during the radiation process. The field is continuously shaped by a multi‐leaf collimator (MLC). The positions of the gantry, the collimator, all leaves and the dose output at any time of the beam delivery are decisive factors for the treatment plan quality. Furthermore, it must be assured that the patient, especially their tumor region, is positioned correctly. This complex radiation technique therefore requires time‐consuming pre‐treatment quality assurance (QA) procedures for all patient plans.

Common methods of pre‐treatment delivery QA are two‐ or three‐dimensional array detectors, such as MapCHECK*®* (Sun Nuclear, Melbourne, FL), ArcCHECK*®* (Sun Nuclear, Melbourne, FL) or Delta^2^ (Scandidos, Uppsala, Sweden). These measurement arrays with diodes or ionization chambers are a time‐consuming approach to check complex VMAT plans^3^, ^4^ and cannot be used during patient treatment.

An alternative method for delivery QA is integrating log file analysis into clinical routine.^2^, ^5^ Log files are automatically generated by the linear accelerator (linac) and contain all relevant plan data. Log files are considered as a practical and time‐saving method as part of patient QA. Haga et al. showed that the calculated dose distribution of the treatment planning system (TPS) plan agreed well with the re‐calculated log file using 2%/2 mm gamma index criteria.^6^ When using log file analysis, machine QA becomes more essential, because all dose relevant parameters in the log file must be checked.^7^ Using only log file analysis for delivery QA without any other physical measurements is thoroughly discussed in the literature.^8^

Numerous delivery QA methods have already been compared with each other.^9^, ^10^, ^11^ However, the comparison of the sensitivity of 4 Hz log files of an Elekta Synergy linac with the sensitivity of a standard three‐dimensional (3D) phantom for VMAT delivery QA has not yet been reported in literature. The aim of this study is to compare log file analysis (LINACWatch*®*, Qualiformed) with a standard 3D phantom (ArcCHECK*®*, Sun Nuclear) and ionization chamber using different kinds of treatment plans containing MLC, dose, and collimator errors.

## 2 MATERIALS AND METHODS

### 2.A Treatment plans and introduced errors

A total of 36 reference plans of different entities (12 prostate, 12 head and neck, 12 SBRT thoracic and head metastasis) were used in this study. Each plan was manipulated using different kinds of errors created by an in‐house Matlab*®* (MathWorks Inc. Natick, MA, USA) script. Four kinds of errors with different magnitudes were applied to the treatment plans. First, misalignments of the MLC were applied (MLC opening and MLC closing from 0.25 to 0.75 mm, increment 0.25 mm). Second, unidirectional MLC shifts of 1 to 3 mm with increment of 1mm were used. Third, collimator errors (+2° and +4°) and finally dose errors (‐4% to +4%, increment of 2%) were implemented. This led to 15 different kinds of errors, whereas one kind of error was included in each plan, leading to 36 error‐free plans (reference plans) and 540 incorrect plans. All 576 plans were irradiated once on ArcCHECK*®* and the respective log file was analyzed by LINACWatch*®*. Both systems compared each plan with the corresponding reference plan.

### 2.B Equipment

#### 2.B.1 Linear accelerator (linac)

All measurements were conducted with an Elekta Synergy® linac equipped with the Agility® head. The positions of the leaves were measured optically. VMAT plans with 6MV photon energy were used in this study.

#### 2.B.2 ArcCHECK® (AC) and ionization chamber

The ArcCHECK*®* detector (Sun Nuclear, Melbourne, FL) is a cylindrical phantom for pre‐treatment delivery QA, specifically designed for rotational deliveries. The outer diameter of the phantom is 26.8 cm and contains a total of 1386 detectors (SunPoint^TM^ diodes), arranged helically with a detector spacing of 1 cm. The corresponding SNC software (Sun Nuclear) Version 6.5 was used to analyze all measurements. The comparison between the TPS plan and the measurement was performed using the gamma index method and a 0.125 cm^3^ PTW (PTW, Freiburg, D) ionization chamber in the isocenter of AC.

#### 2.B.3 LINACWatch® (LW)

**Fig. 1:**
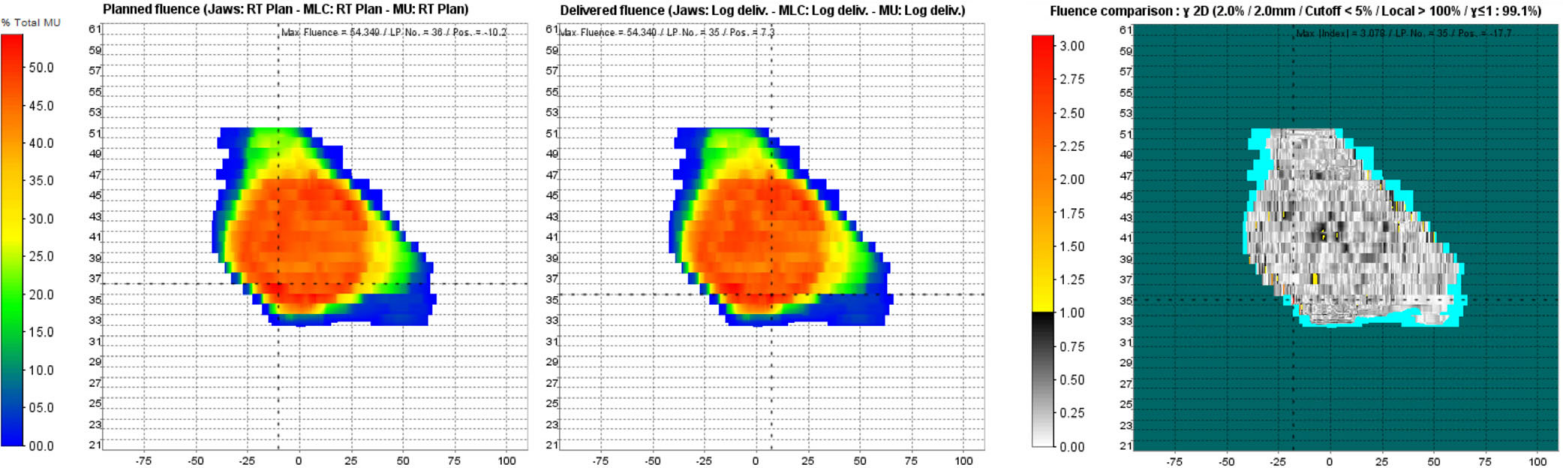
LINACWatch® v3.4.0. GUI. Calculated fluence of treatment planning system (left); Calculated fluence of log file (middle); Fluence comparison using 2%/2 mm gamma criteria (right).

LINACWatch® (Qualiformed, La Roche‐sur‐Yon, FRA) collects and analyzes 4Hz log file data of the Elekta Synergy® linac. It calculates the gamma‐index passing rate between the fluence of TPS and log file (see Fig. 1). Moreover the log file provides the position of each leaf, the gantry and the collimator, as well as the dose output (monitor units, MU). Due to the nature of the fluence calculation used in LW, only delivery errors could be detected. However, errors in TPS calculations (beam modeling or beam data) could not be identified and no points about plan quality could be made.

#### 2.B.4 Treatment planning system (TPS) and treatment plans

Treatment planning was performed with Monaco® 5.11.0.2 (Elekta, Crawley, UK). The VMAT plans were calculated with 6 MV energy and the collimator position was constantly at 45°. For all 12 prostate plans (6 prostate only, 6 prostate including the lymphatic nodes), the gantry delivered one beam composed of two full 360° arcs, clockwise and counterclockwise. For all prostate plans, a fraction dose of 2 Gy was used. The 12 head and neck plans were divided into four radiation plans for patients being treated solely on the left side (two to three arcs between 180° and 220°), four plans on the right side (two to three arcs between 220° and 340°) and four plans for patients being treated with radiotherapy on both sides (two to three arcs between 280° and 360°). For all head and neck plans the fraction dose was between 1.8 and 2 Gy. Additionally, the 12 SBRT plans were divided into 6 thoracic and 6 head metastasis plans. The fractional dose varied between 4 and 10 Gy and the planning target volume was between 100 and 200 cm^3^. The SBRT radiotherapy plans were composed of one to two arcs between 180° and 360°.

### 2.C Statistical analysis and dosimetric comparison

Every plan was irradiated on the ArcCHECK phantom and the corresponding, simultaneously acquired log file was sent to LINACWatch. Thus, the same plan was examined by both delivery QA systems. Sensitivity was defined as the ratio of properly detected incorrect plans to all incorrect plans according to formula (1). For specificity, plans free from any error (reference plans) were taken into consideration according to formula (2).

(1) $$\text{sensitivity}\left(\text{\textbackslash{}\%}\right)=\frac{\text{number of detected non}‐\text{error}‐\text{free plans}}{\text{total number of non}‐\text{error}‐\text{free plans}}\cdot 100$$

(2) $$\text{specificity}\left(\text{\textbackslash{}\%}\right)=\frac{\text{number of detected reference plans}}{\text{total number of reference plans}}\cdot 100$$

Two different protocols for ArcCHECK*®* and two different protocols for LINACWatch*®* were used to detect the incorrect plans. AC is widely used, and standard gamma criteria (see Table 1) can be found in in the literature and were applied for AC evaluation.^10^, ^11^ For LW, no standard gamma criteria have been established yet in the literature. It was important for us that reference plans could be detected as such (specificity = 100%). Due to the nature of the gamma value calculation, applying the same criteria to different systems will not lead to comparable results. Therefore, different gamma criteria are required. We researched suitable gamma criteria with LW prior to this study, which led to stricter gamma criteria as well as stricter acceptance limits for LW (see Table 1).

**Table 1:** Table shows all four protocols used in this examination. DD (dose difference), DTA (distance to agreement), TH (threshold), passing rate (PR), PR LIM (acceptance limit), Coll RMS (RMS value of the collimator position), MU RMS (RMS value of the radiated monitor units).

Protocol DD/DTA/mode	Process	TH of Dmax [%]	PR LIM [%]	Coll RMS[°]	MU RMS [%]
3%/3 mm/global	AC	20	95	—	—
2%/2 mm/global	AC	20	90	—	—
2%/2 mm/global	LW	5	95	1	1
1.5%/1.5 mm/global	LW	5	95	1	1

For LW, three evaluation levels were involved for detecting non‐error‐free plans. First, the calculated fluence of the log file and the fluence of the TPS were compared using the gamma index method.^2^, ^12^ The second evaluation level was the root‐mean‐square (RMS) value of the collimator position and third one was the RMS value of the monitor units. The RMS values are calculated using the deviation between the log file data (every 250 ms) and the corresponding reference values from the TPS (where a linear interpolation between control points is used).

The ionization chamber dose difference is standardized to the measured dose of the error‐free plan [see formula (3)].

(3) $$\text{dose difference}\;\left[\text{\textbackslash{}\%}\right]=\frac{\text{Derror}\;‐\;\text{Dreference}}{\text{Dreference}}\cdot 100$$

The statistical evaluation was performed using SPSS (IBM, NY, USA), for the evaluation of the sensitivity, specificity and the dose of the ionization chamber. A *p*‐value smaller than 0.05 was defined as statistically significant.

## 3 RESULTS

### 3.A Overview — all 576 plans

Table 2 shows the sensitivity of all 540 non‐error‐free plans, depending on the analysis protocol and measurement device.

**Table 2:** Sensitivity of the ArcCHECK*®* phantom and LINACwatch*®* for all protocols used in this study. Errors are divided into four subgroups and different magnitudes. *LW, only gamma criteria method (without the limit of the RMS value of the MU); **LW, only the RMS value of the collimator position used for detection (because gamma criteria are not possible for collimator position).

Protocol	3%/3 mm/global	2%/2 mm/global	2%/2 mm/global	1.5%/1.5 mm/global
	AC	AC	LW	LW
Total sensitivity	**20.4%**	**49.6%**	73.9% (63.0%*)	86.5% (73.3*)
**Total MLC error sensitivity**	**19.0%**	**47.7%**	**50.5%**	**75.5%**
−0.75 mm	27.8%	66.7%	91.7%	97.2%
−0.50 mm	8.3%	33.3%	36.1%	94.4%
−0.25 mm	5.6%	13.9%	19.4%	44.4%
+0.25 mm	5.6%	22.2%	19.4%	33.3%
+0.50 mm	13.9%	55.6%	41.7%	91.7%
+0.75 mm	52.8%	94.4%	94.4%	91.7%
**Total MLC shift error sensitivity**	**29.6%**	**66.7%**	**66.7%**	**83.3%**
+1.00 mm	0%	22.2%	0%	50%
+2.00 mm	16.7%	80.6%	100%	100%
+3.00 mm	72.2%	97.2%	100%	100%
**Total Coll error sensitivity**	**25.0%**	**44.4%**	100.0%**	100.0%**
2°	5.6%	30.6%	100.0%**	100.0%**
4°	44.4%	58.3%	100.0%**	100.0%**
**Total dose error sensitivity**	**13.2%**	**42.4%**	100.0% (59.0%*)	100.0% (92.4%*)
−4%	11.1%	47.2%	100% (100.0%*)	100% (100.0%*)
−2%	2.8%	8.3%	100% (16.7%*)	100% (83.3%*)
+2%	2.8%	25.0%	100% (19.4%*)	100% (86.1%*)
+4%	36.1%	88.9%	100% (100.0%*)	100% (100.0%*)

In general, the results indicate that for tighter gamma criteria, the sensitivity of the system increases. If the gamma criteria are too low, the specificity decreases. Specificity shows no significant differences between all used protocols. All error free plans were detected as such, indicating precise delivery of linac.

The stricter gamma index for AC (2%/2 mm) and LW (1.5%/1.5 mm) with a detection level of >90% and >95% does not ensure finding all MLC positioning, dose or collimator positioning errors used in this study (see Table 1).

LW is more sensitive in detecting the implemented errors introduced in this study. For MLC misalignments, MLC shift and collimator errors, the sensitivity of both LW protocols is higher than the strictest AC protocol (2%/2 mm).

AC device cannot detect all dose error plans up to ±4%. LW could detect all ±2% and ±4% dose errors using the RMS value of the monitor units. Furthermore, using only gamma index criteria, LW can identify all non‐error‐free plans with ±4% dose error.

With the RMS value of the collimator position of the log file, all non‐error‐free plans (collimator error +2° and +4°) can be detected using 1° detection level. With the RMS value of the monitor units, all non‐error‐free plans (dose error ±2% and ±4%) can be detected using 1% detection level. The RMS value of the collimator position and the RMS value of the radiated monitor units are illustrated in Table 3.

**Table 3:** RMS value from the log file of the collimator position and the RMS value of the radiated monitor units are represented.

Error type	Magnitude of error	LW RMS values	
		Coll (°)	MU (%)
No error	—	0.07 ± 0.06	0.21 ± 0.25
Collimator	+2°	2.02 ± 0.07	—
	+4°	3.95 ± 0.10	—
Dose	‐4%	—	2.31 ± 0.07
	‐2%	—	1.18 ± 0.05
	+2%	—	1.17 ± 0.07
	+4%	—	2.29 ± 0.06

### 3.B Individual analysis: prostate — H&N — SBRT

In general, steeper curves indicate higher sensitivity of the protocol or system. The figures show the response of the gamma value of the respective system to implemented errors.

#### 3.B.1 MLC misalignments

**Fig. 2:**
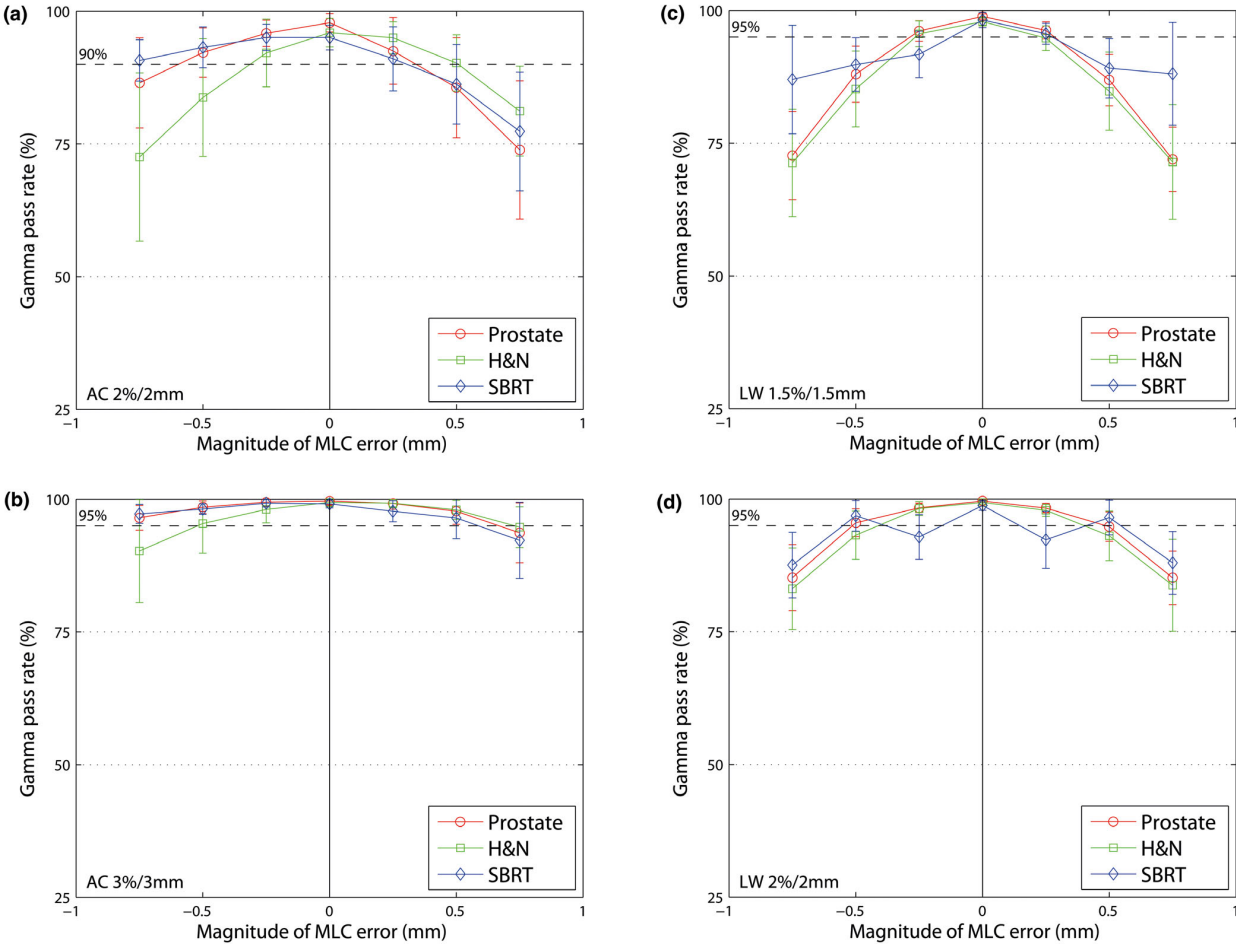
Mean gamma passing rate (±1 SD) for three entities (prostate, H&N and SBRT). (a) AC 2%/2 mm, (b) AC 3%/3 mm, (c) LW 1.5%/1.5 mm and (d) LW 2%/2 mm. The dashed line shows the limit for each protocol. MLC misalignments (opening and closing) with different magnitudes (‐0.75 to +0.75 mm, increment of 0.25 mm) are illustrated.

Figure 2 shows the mean gamma passing rate depending on the magnitude of the MLC misalignments for all treatment sites and protocols. Both AC and LW show a symmetrical decrease of the gamma passing rate to closing and opening MLC errors. It shows that the higher the magnitude of the error, the lower the gamma values.

#### 3.B.2 MLC shift errors

**Fig. 3:**
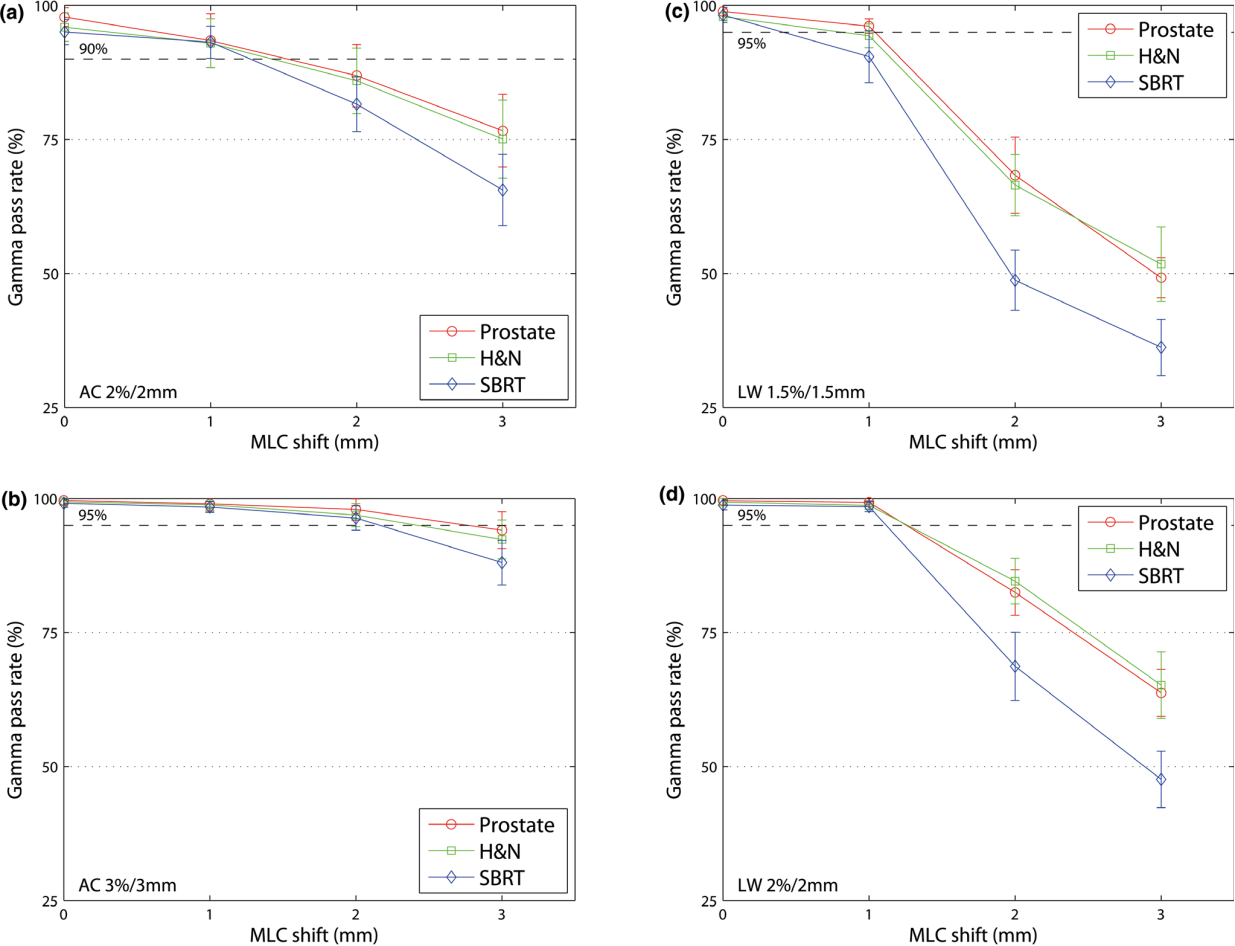
Mean gamma passing rate (±1 SD) for three entities (prostate, H&N and SBRT). (a) AC 2%/2 mm, (b) AC 3%/3 mm, (c) LW 1.5%/1.5 mm and (d) LW 2%/2 mm. The dashed line shows the limit for each protocol. MLC shift errors with different magnitudes (0 to +3.00 mm, increment of 1.00 mm) are illustrated. LW is more sensitive in detecting an MLC shift error than AC.

Figure 3 shows the mean gamma passing rate depending on an MLC shift error. The sensitivity of all systems for detecting an MLC shift error is lower in prostate and H&N than in SBRT plans. SBRT behavior differs from prostate and H&N, because of its smaller field size. Therefore, the impact of implemented errors is higher in SBRT. LW is more sensitive in detecting an MLC shift error than AC using the prescribed protocols.

#### 3.B.3 Collimator errors

**Fig. 4:**
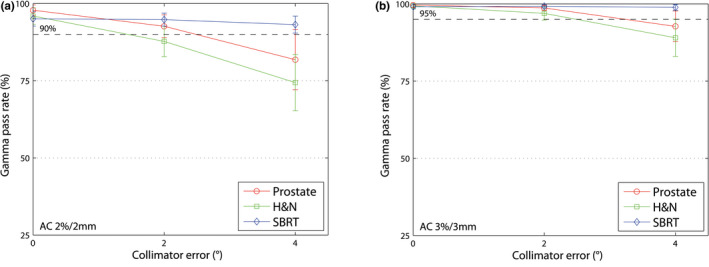
AC, mean gamma passing rate (±1 SD) for different collimator errors up to 4°. (a) 2%/2 mm gamma passing rate and (b) 3%/3 mm gamma passing rate.

Figure 4 shows the gamma passing rate depending on the collimator position. Due to field size, the AC device is more sensitive for H&N than for prostate and SBRT. AC (2%/2 mm and 3%/3 mm) could detect 4° collimator errors for prostate plans. For SBRT plans, AC could not detect errors up to 4°. Collimator errors could be detected from 1° with LW by the RMS value.

#### 3.B.4 Dose errors

**Fig. 5:**
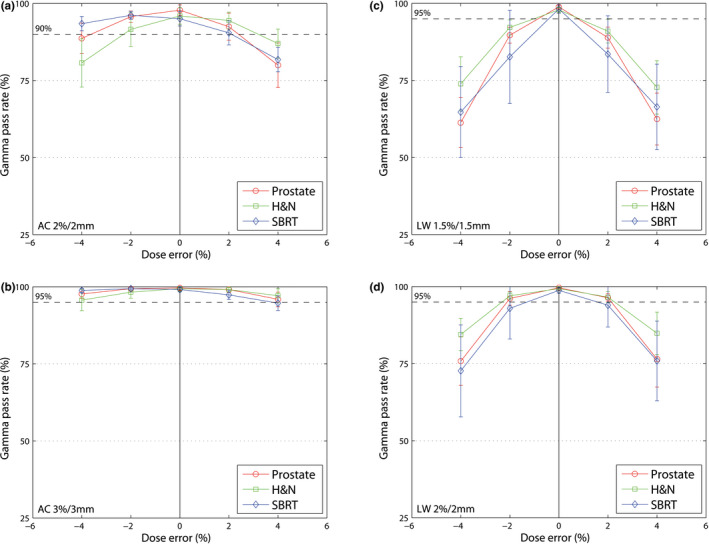
Mean gamma passing rate (±1 SD) for three entities (prostate, H&N and SBRT). (a) AC 2%/2 mm, (b) AC 3%/3 mm, (c) LW 1.5%/1.5 mm and (d) LW 2%/2 mm. The dashed line shows the limit for each protocol. Dose errors with different magnitudes (‐4% to +4%, increment of 2%) are illustrated.

**Fig. 6:**
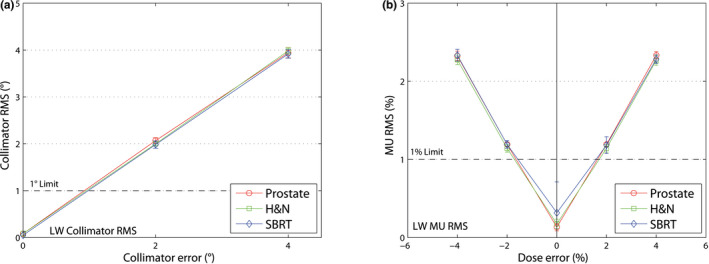
Logfile data from LW, (a) mean RMS value (±1 SD) of the collimator position for collimator errors up to 4°. (b) mean RMS value (±1 SD) of the monitor units for dose errors up to ±4%. The limits (dashed line) were set to 1° and 1%, respectively.

LW shows a higher sensitivity than AC device (see Fig. 5). LW (1.5%/1.5 mm) could detect all dose errors from ±2% with the fluence gamma criterion and additionally, the same errors with the RMS value of the monitor units set to 1% limit (see Fig. 6).

#### 3.B.5 Ionization chamber — effect on dose

**Fig. 7:**
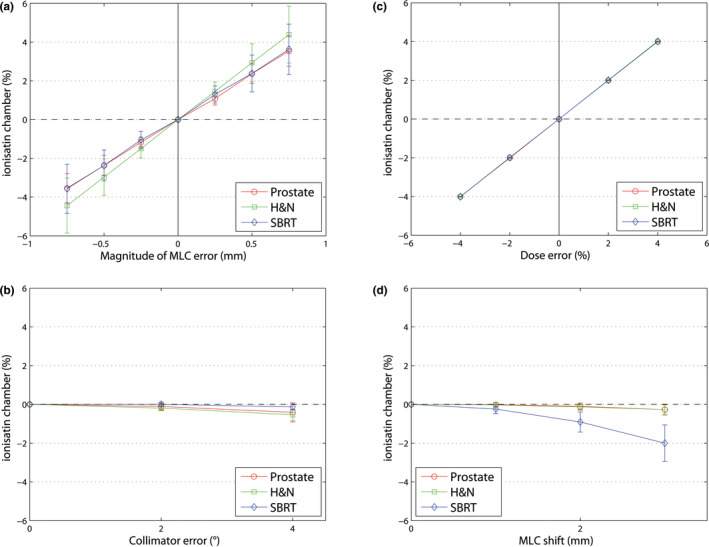
Mean dose difference between the reference plans of the ionization chamber (±1 SD) at the isocenter of the AC for three entities (prostate, H&N and SBRT). (a) MLC opening/closing errors, (b) collimator position errors, (c) dose errors, and (d) MLC shift errors.

The measured dose (0.125 cm^3^ ionization chamber) shows strong correlation and dose effects to the magnitude of an MLC opening/closing (different field size) for all entities. For H&N plans, field size changes have the biggest influence on the dose. Collimator errors up to 4° have hardly any influence on the measured dose at isocenter of the AC. For MLC shift error, the dose output varies for the three entities. For SBRT plans, the influence on the isocenter dose of the AC is biggest because of the small field size (see Fig. 7).

## 4 DISCUSSION

In our study, all reference plans were correctly detected as plans without any error for all protocols used. Both examined gamma criteria for each system (AC and LW) were applicable for clinical use in terms of specificity. Gamma values of reference plans were higher for LW than for AC, which led to stricter gamma criteria as well as stricter acceptance limits (e.g. LW 1.5%/1.5 mm had a higher passing rate for the reference plans than AC 2%/2 mm). For less strict criteria applied on LW, the sensitivity would have declined, and the specificity would have remained constant. Consequently, less strict criteria for LW would not lead to any advantage. This is the opposite for AC: A stricter criteria led to a decline in specificity.

There is a strong correlation between the gamma passing rate and the magnitude of the errors in the treatment plans. The passing rate responded stronger to errors in case of log file analysis. For all treatment sites (prostate, H&N and SBRT) these results clearly show that log file analysis is more sensitive than AC measurements regardless of the applied protocol used in this study.

The agreement for both the reference and the non‐error‐free plans for AC gamma passing rate is consistent with previous research not using log files.^3^, ^9^, ^10^, ^11^, ^13^, ^14^ Many studies evaluated the sensitivity of different QA methods by including different kinds of errors. For example, MLC misalignments errors were investigated by Masahide Saito et al. with different QA methods such as Delta 4.^15^ The results obtained when trying to detect MLC errors of our AC protocols are comparable with the results of their study. Delta 4 shows the same behavior as the ArcCHECK Phantom for both global gamma protocols with 3%/3 mm and 2%/2 mm.

Moliner et al. investigated, among other QA tools, the AC detector. The results of this study, using collimator, dose and MLC errors, are in agreement with our investigation of gamma criteria 3%/3 mm and 2%/2 mm for all treatment sites.^10^

Log files are generated by the linac and therefore are insensitive to miscalibration of any component such as leaf position. Pay attention to the fact that when using fluence calculations for log files, only delivery errors could be detected, but no TPS errors or bad plan quality. In their study, Agnew A. et al. discussed that it is necessary to increase linac‐specific QA when using log files for delivery QA.^7^ Norvill et al. recommended using a machine QA tool with submillimeter accuracy for the position of the MLC for intensity modulated radiotherapy.^16^

Pre‐treatment delivery QA must be performed for every patient due to the complexity of modulated treatment techniques. An advantage is that log files are generated automatically during every treatment and can be used for every single fraction to analyze the correct delivery of treatment plans. Log file analysis with LW is a convenient and time‐saving QA tool to find such delivery errors. Kabat et al. demonstrated that log files can increase the efficiency of QA for Elekta linacs.^5^

The essential strength of our investigation is that all measurements were performed with AC, LW and ionization chamber at the same time. Log files were generated during AC measurements of all plans. Further strengths are the use of different treatment sites (prostate, H&N and SBRT) and various fractional doses.

These methods could be further be refined by also considering the high‐resolution log files (25 Hz instead of 4 Hz). Fluence without CT data does not illustrate the clinical consequences of an irradiation error, because dose recalculation is not performed. Therefore, no TPS data (beam modeling, beam data) or plan quality is checked. More accurate calculation algorithms for log file analysis, such as an independent Monte Carlo (in combination with CT), could increase the efficiency of detecting clinically relevant errors. Furthermore, it enables finding different kinds of TPS errors, especially beam data or beam modeling.

## 5 CONCLUSION

Our conclusion is that log file analysis is an excellent tool for delivery QA with Elekta linacs of VMAT plans. LW is very sensitive to detect small delivery errors. We recommend using LW with 1.5%/1.5 mm global for the gamma calculation delivery QA. We also recommend using RMS limits of 1° for collimator position and 1% for dose errors. Log file analysis is an outstanding complement to phantom‐based delivery QA, which, consequently, we integrated successfully into our clinical routine.

## CONFLICT OF INTEREST

The authors have no relevant conflict of interest to disclose.
